# CD44 Promotes Intoxication by the Clostridial Iota-Family Toxins

**DOI:** 10.1371/journal.pone.0051356

**Published:** 2012-12-07

**Authors:** Darran J. Wigelsworth, Gordon Ruthel, Leonie Schnell, Peter Herrlich, Josip Blonder, Timothy D. Veenstra, Robert J. Carman, Tracy D. Wilkins, Guy Tran Van Nhieu, Serge Pauillac, Maryse Gibert, Nathalie Sauvonnet, Bradley G. Stiles, Michel R. Popoff, Holger Barth

**Affiliations:** 1 Integrated Toxicology Division, Medical Research Institute of Infectious Diseases, Frederick, Maryland, United States of America; 2 Core Imaging Facility, University of Pennsylvania School of Veterinary Medicine, Philadelphia, Pennsylvania, United States of America; 3 Institute of Pharmacology and Toxicology, University of Ulm Medical Center, Ulm, Germany; 4 Leibniz Institute for Age Research, Fritz Lipmann Institute, Jena, Germany; 5 Laboratory of Proteomics and Analytical Technologies, National Cancer Institute, Frederick, Maryland, United States of America; 6 TechLab, Inc., Blacksburg, Virginia, United States of America; 7 Department of Intracellular Communications and Infectious Microorganisms, College of France, Paris, France; 8 Institut Pasteur, Unité des Bactéries anaérobies et Toxines, Paris, France; 9 Institut Pasteur, Unité de Biologie des Interactions Cellulaires, Paris, France; 10 Biology Department, Wilson College, Chambersburg, Pennsylvania, United States of America; Universidad de Costa Rica, Costa Rica

## Abstract

Various pathogenic clostridia produce binary protein toxins associated with enteric diseases of humans and animals. Separate binding/translocation (B) components bind to a protein receptor on the cell surface, assemble with enzymatic (A) component(s), and mediate endocytosis of the toxin complex. Ultimately there is translocation of A component(s) from acidified endosomes into the cytosol, leading to destruction of the actin cytoskeleton. Our results revealed that CD44, a multifunctional surface protein of mammalian cells, facilitates intoxication by the iota family of clostridial binary toxins. Specific antibody against CD44 inhibited cytotoxicity of the prototypical *Clostridium perfringens* iota toxin. Versus CD44^+^ melanoma cells, those lacking CD44 bound less toxin and were dose-dependently resistant to *C. perfringens* iota, as well as *Clostridium difficile* and *Clostridium spiroforme* iota-like, toxins. Purified CD44 specifically interacted *in vitro* with iota and iota-like, but not related *Clostridium botulinum* C2, toxins. Furthermore, CD44 knockout mice were resistant to iota toxin lethality. Collective data reveal an important role for CD44 during intoxication by a family of clostridial binary toxins.

## Introduction

Some pathogenic *Clostridium* and *Bacillus* species produce structurally and functionally related binary protein toxins. Amongst the clostridia, binary toxins are produced by *Clostridium botulinum* (C2), *Clostridium difficile* (CDT), *Clostridium perfringens* (iota), as well as *Clostridium spiroforme* (CST) [1]–[3]. These toxins consist of distinct proteins (A and B) not linked in solution and respectively possess ADP-ribosyltransferase, as well as cell-binding/membrane translocation, properties [2], [4]–[7]. Upon cytosolic entry, A-components mono-ADP-ribosylate globular (G)-actin at arginine-177 that then inhibits actin filament formation and destroys the cytoskeleton, ultimately rounding cells [2]. Iota, CDT, and CST toxins represent the iota family that share high sequence homology (81% identity among B components), form functional inter-species chimeras, and are cross-neutralized by heterologous antibody [1]–[3]. In contrast, C2 toxin does not form biologically-active chimeras with any iota-family components. The B components of iota-family and C2 toxins share only 44% sequence identity, and the latter uniquely binds to asparagine-linked carbohydrates on an unidentified cell-surface protein [8], [9]. Recent reports reveal that lipolysis-stimulated lipoprotein receptor (LSR) is a cell-surface receptor for *C. difficile* CDT, *C. perfringens* iota toxin, and *C. spiroforme* CST [10], [11]. In contrast, *C. botulinum* C2 toxin does not bind LSR [10].

These binary toxins form complexes on targeted cells after release from the bacterium as separate proteins [1], [2], [12]–[17]. B components initially bind to the cell surface, either as monomer or ring-shaped homo-heptamers formed in solution, and the A components dock to B components on the cell surface. These AB complexes are internalized into endosomes, followed by A component(s) release into the cytosol via pores formed by B heptamers under acidic conditions [2], [12], [14]–[18].

Previous studies reveal that the protease-activated B component of iota toxin (Ib) associates with lipid rafts on Vero cells [14], [17] via a pronase-susceptible protein not affected by other proteases, lipases, or lectins [13]. To facilitate discovery of potential proteins involved in the intoxication process, there was quantitative ^18^O/^16^O-based proteomic profiling of lipid rafts isolated from Vero cells incubated with, and without, Ib [19]. Results revealed ninety different proteins with increased relative concentrations in lipid rafts from cells incubated with Ib. One of the proteins most highly enriched in Ib-containing rafts was CD44, a type I cell-surface glycoprotein involved in diverse functions among different cell types [20], [21]. We performed a series of experiments with cultured cells, as well as animals, to investigate whether CD44 is involved in the mode of action of clostridial binary toxins. Results implicate a role for CD44 during intoxication by the iota-family toxins.

## Results

### Reducing Agent or Antibody Against CD44 Inhibits Iota Cytotoxicity

**Figure 1:**
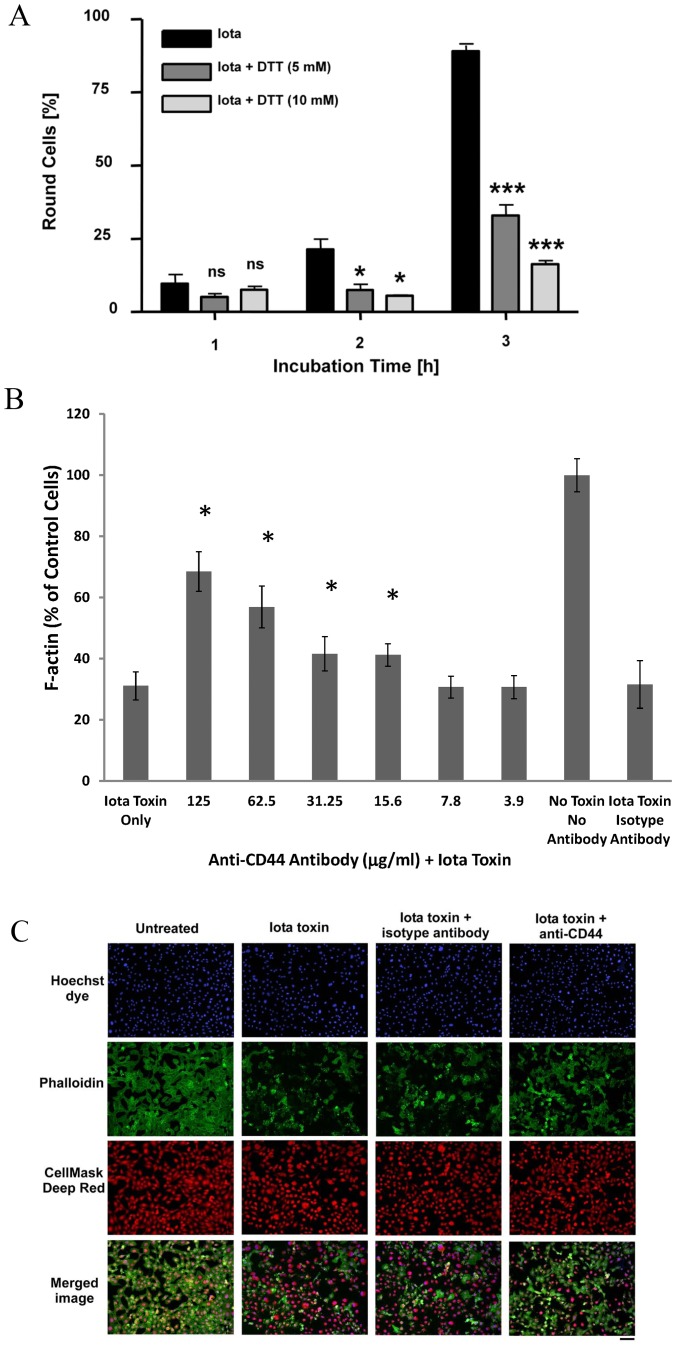
Effects of DTT upon iota cytotoxicity. (**A**) DTT (5 and 10 mM) effects upon iota (460 pM Ia +500 pM Ib) cytotoxicity with Vero cells (n = 3 wells per calculation). “*” and “***” represent significant differences at p<0.05 and p<0.0005, respectively. ns = not significant. (**B**) Inhibition of iota cytotoxicity with anti-CD44 antibody. Vero cells were pre-treated with serial dilutions of an anti-CD44 monoclonal antibody for 30 min before adding iota toxin. A non-specific isotype antibody was included as a control. After 4 h of intoxication, the actin cytoskeleton was stained with Alexa-488 phalloidin. Images were acquired and integrated fluorescence intensity of Alexa-488 phalloidin staining determined with MetaXpress software. Values represent the mean of nine fields +/− standard deviation. “*” designates statistically different (ANOVA p<0.05) than isotype control antibody plus iota toxin, or toxin only. (**C**) Typical visual results showing Vero cells following various treatments and subsequent staining with Hoechst (nucleus), Alexa-488 phalloidin (F-actin), and CellMask Deep Red (cytoplasm and nucleus).

Disulfide-driven clustering of CD44 on the cell surface promotes binding of a natural ligand (hyaluronan) to cells and is inhibited by a reducing agent like dithiothreitol (DTT) [22]. As iota toxin also forms oligomers on Vero and MDCK cells [14], [16], [17], [23], and CD44 was our top proteomics-based candidate involved in intoxication of Vero cells, we first examined if DTT had any overt effect upon iota intoxication. Figure 1A shows that either 5 or 10 mM DTT significantly delayed overt rounding due to iota activity, versus cells incubated with toxin alone. However, by 12 h the DTT-treated Vero cells eventually rounded due to iota toxin. In contrast, Vero cells incubated with high picomolar concentrations of C2 toxin were not protected by 10 mM DTT (data not shown). Control cells incubated with either 5 or 10 mM DTT alone showed no change in morphology.

Regarding the effects of DTT upon each component of iota toxin, we first excluded that DTT (10 and 50 mM) interfered with A component (Ia)-catalyzed ADP-ribosylation of actin from Vero cell lysate *in vitro* (data not shown) [7]. Furthermore, Ib binding to cells was not inhibited by DTT (10 mM) as determined by Western blot analysis (data not shown). In conjunction with the earlier proteomic-based findings [19], sensitivity to DTT suggested that CD44 might play a role in iota cytotoxicity; however, the non-specific nature of this reducing agent upon any disulfide-containing protein found on a cell surface required more targeted experimentation.

Experiments were next done to neutralize the cytotoxic effects of iota toxin via a specific monoclonal antibody against CD44 (Figs. 1B and 1C). Pretreatment of Vero cells with this antibody afforded protection against iota intoxication, as evidenced by inhibited rounding and disruption of the actin cytoskeleton. The latter was quantitatively measured by binding of fluor-labeled phalloidin to filamentous (F) actin. Figure 1B summarizes the dose-dependent, protective effect of anti-CD44 upon iota intoxication with representative images in Figure 1C. With nine fields per sample, the mean readings of cells incubated with iota toxin and varying antibody concentrations (15.6–125 µg/ml) were significantly different versus controls consisting of: 1) isotype control antibody plus iota toxin; or 2) iota toxin only. These results strongly suggested a crucial role, whether direct or indirect, for CD44 during intoxication of cells by iota toxin.

### CD44-deficient Cells are Resistant to Iota-family Toxins

**Figure 2:**
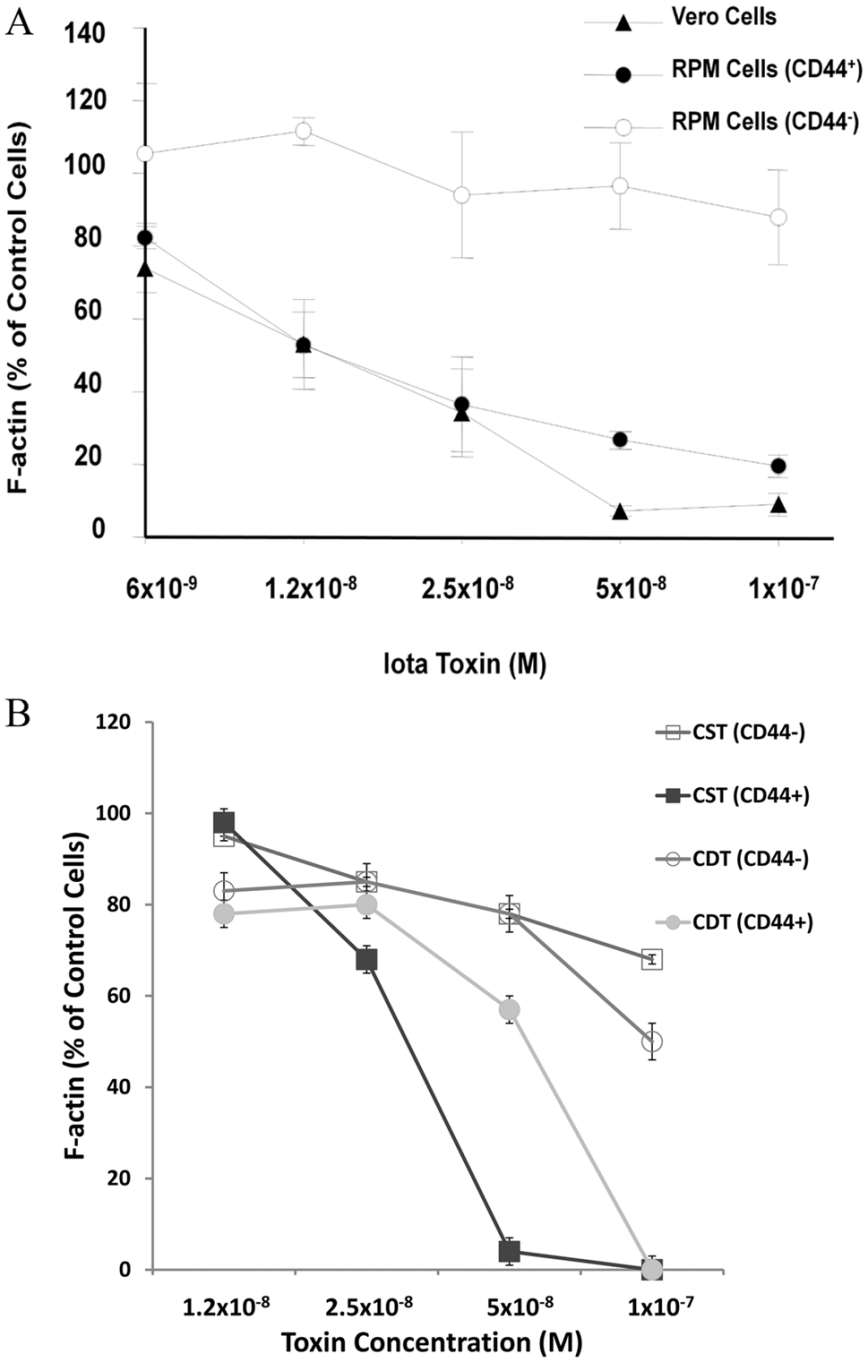
CD44^−^ cells are resistant to iota and iota-like toxins versus CD44^+^ cells. (**A**) Dose-response of iota toxin on cells with controls consisting of cells in media only. The Y-axis represents the “% control” of F-actin content (Alexa-488 phalloidin stained after 5 h) in intoxicated cells versus controls in media only. (**B**) Like iota toxin, CD44^+^ RPM cells are also susceptible to *C. difficile* (CDT) and *C. spiroforme* (CST) binary toxins. Each assay was done in duplicate and values represent mean +/− standard deviation from three separate experiments.

**Figure 3:**
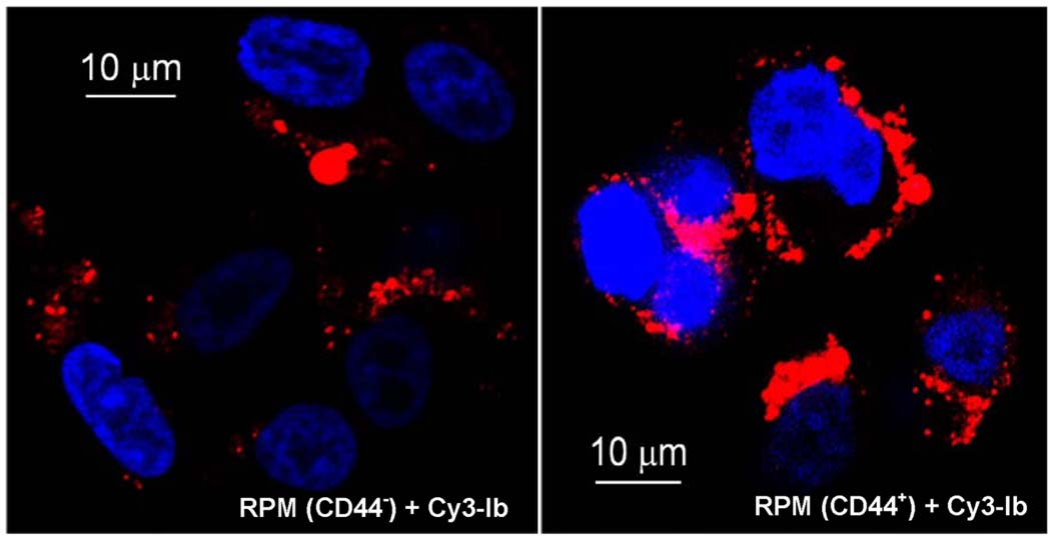
Comparative binding of Ib to CD44^+^, versus CD44^−^, cells. Confocal microscopy was done with RPM (CD44^−^ and CD44^+^) cells incubated with Cy3-labeled Ib (10^−7^ M in DMEM +0.1% BSA) for 3 min at 37°C, washed with PBS, and mounted in mowiol. Blue represents Dapi-stained nuclei and red indicates cell-bound Ib. A representative field of cells is shown.

To further discern the role of CD44 during intoxication by iota-family toxins, cell-based investigations were initiated with a transfectable, CD44-deficient line and varying concentrations of iota toxin (Fig. 2). A human recurrent cutaneous melanoma (RPM) [24] lacking CD44 was resistant to iota toxin over a broad concentration range; however, these same cells following transfection with the CD44 gene importantly became susceptible to these same concentrations of iota toxin (Fig. 2A). The CD44 status of these cells was confirmed by Western blot (data not shown). These results indicated that CD44 plays a role during intoxication of cells with iota toxin. The susceptibility pattern of CD44^+^ RPM cells to iota toxin was very similar to Vero cells commonly used for cytotoxicity assays (Fig. 2A). When higher concentrations (10^−7^ M) of iota toxin were incubated with non-transfected RPM cells, there was some cytotoxicity. Further confirmation of these findings was evident by confocal microscopy showing that Cy3-labeled Ib more readily bound to the CD44^+^, versus CD44^−^, RPM cells (Fig. 3). These results demonstrated that reconstitution of CD44 expression in RPM cells increased binding, and subsequent cytotoxic effects, of iota toxin.

Prompted by these observations with iota toxin, it was important to examine if other closely related binary toxins produced by *C. difficile* and *C. spiroforme* similarly affected the CD44^+/^CD44^−^ RPM cells. Results (Fig. 2B) clearly revealed that these iota-like toxins had similar cytotoxicity patterns as iota toxin. Interestingly, RPM cells were resistant to *C. botulinum* C2 intoxication, independent of CD44. The reason underlying this resistance is not clear; however, cumulative results revealed an essential role for CD44 during intoxication by iota-family toxins.

As LSR has been recently identified as a receptor for iota-family toxins [10], [11], it was important to determine if: 1) LSR is present on RPM cells; and 2) if present, does LSR interact with CD44 as evidenced by co-precipitation. The RPM (CD44^+^ or CD44^−^) as well as Vero cells [10] do contain LSR as determined by Western blot analysis; however, there is no detectable interaction between LSR and CD44 (with or without added Ib) in co-precipitation experiments (data not shown).

### Iota-family Toxins Bind to Purified CD44

**Figure 4:**
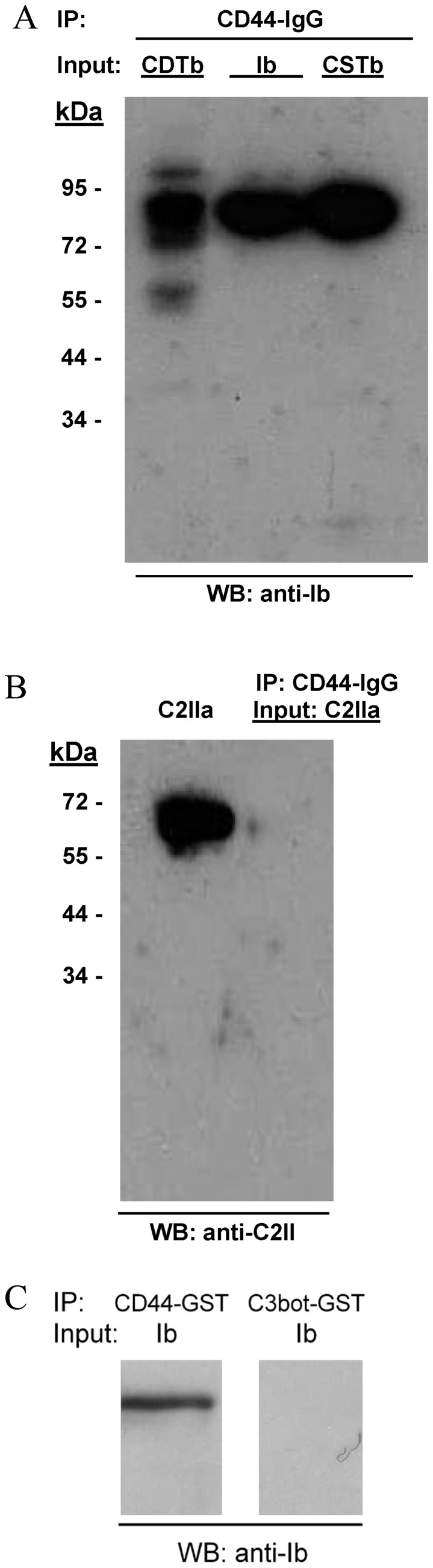
Binding of iota-family B components to purified CD44 in solution. (**A and B**) The B component (10 µg) of each toxin was added to CD44-IgG (10 µg in 50 µl) at room temperature for 60 min. Protein A - agarose beads were then added for 5 min at room temperature, gently centrifuged, and washed with buffer. SDS-PAGE sample buffer containing reducing agent was added to the beads, the mixture heated, and protein separated from the beads by centrifugation. Supernatant proteins were then resolved on a 10% gel, transferred onto nitrocellulose, and clostridial B component detected with either 1∶1000 dilutions of rabbit anti-Ib (Panel A) or anti-C2II sera (Panel B). Protein A - peroxidase conjugate was used to detect bound antibody, and following washes, specific bands were visualized with chemiluminescent substrate. **(C)** Like the CD44-IgG construct, Ib also binds specifically to a CD44-GST construct. A GST version of *C. botulinum* C3 exoenzyme, used as a negative control, does not bind to Ib in pull-down experiments done similarly for panels A and B, with an exception being the use of glutathione-sepharose (instead of Protein A-agarose) beads.

To ascertain if CD44 directly binds to iota family toxins, subsequent solution-based, pull-down experiments were done with *C. perfringens* Ib and equivalent molecules produced by *C. spiroforme* and *C. difficile* (Fig. 4A). These B components share ∼80% sequence identity with Ib, and all bound to a CD44-IgG fusion in these experiments. In contrast the B component of *C. botulinum* C2 toxin (C2IIa), which shares only 44% identity with Ib and binds to an asparagine-linked carbohydrate not recognized by iota toxin [8], did not bind CD44-IgG (Fig. 4B). Additionally, to rule out non-specific binding, Ib did not bind to protein A agarose beads or irrelevant IgG in the pull-down experiments (data not shown).

To further confirm these results, pull-down experiments were next done with a CD44-GST fusion. Ib bound to CD44-GST but not an irrelevant GST control linked to *C. botulinum* C3 exoenzyme (Fig. 4C). Overall, these findings with different CD44 constructs clearly revealed specific binding of the iota-family toxins to CD44. The C2 toxin did not bind CD44.

### CD44 Knockout Mice are Resistant to Iota Toxin

To complement the above data, we investigated the lethal effects of iota toxin in CD44 knockout mice versus the parental wild-type. Many clostridial toxins, that includes iota, are lethal in mice following intraperitoneal injection [25], [26]. Upon giving 10 pmoles of Ia (0.5 µg) and Ib (0.75 µg) per animal, there was 100% lethality among wild-types (n = 9) that died within 4 h. In contrast, there was only 12.5% lethality among CD44 knockouts (n = 8).

In conclusion, the collective data consisting of mouse lethality and cell-based cytotoxicity revealed that CD44 facilitates intoxication by the iota-family toxins produced by various pathogenic clostridia.

## Discussion

This current study was made feasible by a lipid-raft based, shotgun quantitative proteomic analysis of Vero cells [19] revealing a potential role of CD44 during intoxication by iota-family toxins from clostridia. In contrast, current results revealed that the related C2 toxin of *C. botulinum* does not use CD44 for intoxication. Upon comparing lipid rafts of Ib-treated cells, versus controls from cells incubated in media only, CD44 was most highly enriched among ninety possible candidates. It is important to note intriguing biological correlations that exist between CD44 and iota toxin. For example, CD44 associates with lipid rafts in epithelial cells, forms cell-surface clusters, is basolaterally located in polarized cells, and use of a cholesterol extracting agent (methyl-β-cyclodextrin) disperses CD44 throughout the cell surface [21]. Discovery that *C. perfringens* iota toxin also associates with lipid rafts [14], [17], rapidly forms temperature-dependent clusters on cells [16], preferentially intoxicates polarized cells through the basolateral surface [18], [27], and that methyl-β-cyclodextrin reduces Ib binding, oligomer formation and delays iota cytotoxicity [17] remarkably parallels the findings for CD44. Upon internalization by a clathrin-independent mechanism, CD44 traffics through an acidified endosome and recycles back to the cell surface, like that reported for clostridial binary toxins [27]–[32]. Altogether, the similar biological aspects of CD44 and iota toxin, along with proteomic-based clues from lipid rafts, logically provided enticing evidence for us to pursue this current study.

Furthermore, pre-treatment of Vero cells with pronase effectively prevents Ib binding and thus suggests a protein-based receptor [13]. It is known that membrane-bound CD44 is susceptible to proteolysis, as evidenced by a membrane-associated metalloprotease that promotes metastasis [33]. Cells incubated with DTT also do not bind hyaluronan through CD44 [22] after reduction of a disulfide bond within the transmembrane domain of CD44 [34]. Modification of select cysteines prevents CD44 dimerization and clustering into lipid rafts [35]. Our initial experiments also showed that pre-treatment of Vero cells with DTT transiently prevented iota intoxication, but this is not due to inhibiting either Ia modification of actin or Ib binding to the cell surface.

For various cell types, CD44 plays a remarkably multi-faceted role that includes surface receptor for multiple ligands (i.e. fibronectin [36], chondroitin sulfate [37], osteopontin [38], hyaluronan [39], heparin-binding growth factor [40]) and signal transducer [20], [41], [42]. There are ten isoforms of CD44 varying within the extracellular stem that become alternatively spliced at the gene level [20]. In this current study, we used the standard form of CD44 that is commonly used in other studies.

The C-terminal cytoplasmic domain of CD44 is linked to the actin cytoskeleton via the ezrin, radixin, and moesin (ERM) family of proteins important for rearranging lipid rafts, filopodia formation, as well as cell migration and overall shape [29], [43]–[45]. Binding of the ERM complex to CD44 is regulated by protein kinase C phosphorylation of Ser 291 on CD44, which has a direct effect upon ezrin interaction with CD44 and ultimately chemotaxis. CD44 (like CD90) is a common cargo protein endocytosed by clathrin-independent carriers from the leading membrane edge of migrating fibroblasts [29].

In addition to ERM, CD44 also complexes with a sodium-proton pump that acidifies the microenvironment and subsequently activates hyaluronidase-2 plus cathepsin B [28]. This same proton pump might promote pH-driven translocation of iota-family enzyme components from the endosome into the cytosol [1], [18], [31], [32]. The pH requirements for cytosolic entry from acidified endosomes differ between the C2 and iota toxins [31], [32], as the latter requires a lower pH perhaps linked to the CD44-proton pump complex. Although there is no literature supporting a co-association between LSR and CD44, it is also possible that these proteins co-facilitate entry of iota-family toxins into cells via an unknown mechanism.

Following Rho-dependent entry into the cytosol via acidified endosomes, clostridial binary toxins destroy the actin-based cytoskeleton through mono-ADP-ribosylation of G actin [1], [2], [4], [5], [31]. This is readily visualized in Vero cells that become quickly rounded following incubation with picomolar concentrations of iota toxin. Interestingly, intracellular concentrations of F actin modulate cell-surface levels of CD44 in osteoclasts [46]. Perhaps as the iota-family toxins disrupt F actin formation, these toxins are prevented from non-productively binding to intoxicated cells containing a disrupted actin cytoskeleton via decreased surface levels of CD44.

Many groups have investigated the various roles played by CD44 in cell biology. However, until now no one has described CD44 as playing a biological role for any clostridial toxin. Our findings now reveal a family of clostridial binary toxins, associated with enteric disease in humans and animals, that exploit CD44. Interestingly, CD44 indirectly affects internalization of the binary lethal toxin of *Bacillus anthracis* into RAW264 macrophages through a β1-integrin complex; however, CD44 does not act as a cell-surface receptor [47]. The lethal and edema toxins of *B. anthracis* clearly share many characteristics with clostridial binary toxins [1], [12], which now include exploiting CD44 during the intoxication process.

In addition to CD44 and identified protein receptors for entry of *Clostridium* and *Bacillus* binary toxins [10], [11], [12], [47], clostridial neurotoxins (botulinum and tetanus) use multiple cell-surface proteins and gangliosides for entry into neurons [48]. Like CD44 described in our current study, the receptors/co-receptors for clostridial neurotoxins are also located in lipid rafts. Although once inside a cell the internal modes of action may differ, various clostridial and bacillus toxins use common cell-surface structures (i.e. lipid rafts) to gain entry into diverse cell types.

The complex interplay between CD44 and LSR during intoxication by the iota-family toxins perhaps involves a similar, yet unique, mechanism as that previously described for the clostridial neurotoxins or *B. anthracis* toxins [10], [11], [12], [47], [48]. To help determine if CD44 and LSR interact on RPM (CD44^+^) and Vero cells, results from co-precipitation experiments yielded no detectable interactions with (or without) added Ib. However, we can not exclude that weak interactions between CD44 and LSR might not be detected by this common experimental procedure. Understanding how CD44 and LSR might work together to internalize the iota-family toxins clearly represents a broad arena for future study. It is possible that like the paradigm proposed for the *B. anthracis* toxins [47], perhaps integrins activated by CD44 affect LSR binding and/or internalization of iota-family toxins?

Whether interactions are direct or indirect, it is clear from other studies that not only bacterial toxins but also various pathogens exploit CD44. Direct attachment of bacteria to a mammalian cell surface and/or the internalization mechanism for intracellular pathogens can be CD44 mediated as evidenced by studies with *Escherichia coli*, *Listeria monocytogenes*, *Shigella flexneri*, and *Streptococcus pyogenes* [49]–[52]. Poliovirus can also use CD44 indirectly for entering host cells. A monoclonal antibody against CD44 blocks poliovirus binding to cells via steric hindrance of CD155, the known cell-surface receptor that physically associates with CD44 [53], [54]. It is possible that CD44 alters LSR conformation on the cell surface, via direct or indirect interactions, which in turn affects binding of the iota-family toxins to a cell. Interactions between CD44 and LSR might also affect pore formation and trafficking of ADP-ribosyl transferase (A component) from the endosome into the cytosol. The effects of receptor on pore formation for *B. anthracis* binary toxins have been described by Pilpa et al. [55]. Again, we could not detect a physical link between CD44 and LSR from cell membranes (with or without Ib) using a standard co-precipitation method.

Our collective data (anti-CD44 antibody with Vero cells, CD44^−/^CD44^+^ RPM cells, as well as CD44 knockout mice) suggest a complex picture for cell binding/entry of iota-family toxins. This has been confirmed by recent studies revealing a common cell-surface receptor (LSR) used by iota-family toxins [10], [11]. Multiple methods of entering cells are not unusual for bacterial toxins, as evidenced by the related binary toxins produced by *B. anthracis* that target unique cell-surface receptors (TEM8 and CMG2) differing ∼1000-fold in binding affinity for PA [12], [56]. Like LSR and CD44, the receptors for *B. anthracis* toxins are also type I transmembrane proteins. It is possible that LSR and CD44 respectively represent high and low affinity receptors for iota-family toxins. However, there are no obvious sequence homologies between the extracellular amino-terminal domains of LSR and CD44 to help explain these single-pass, type I transmembrane proteins as cell-surface receptors for iota-family toxins. Furthermore, studies by others with CD44 knockout mice suggest an alternative receptor(s) to CD44 for hyaluronan binding to cells [57]. Studies with *Shigella* also reveal that anti-CD44 antibody, as well as use of CD44^−^ cells (the same described in this current study), diminish but do not entirely prevent uptake of this bacterium [24].

For this current study it was very important to complement the cell-based and purified CD44 efforts with animal (mouse) experiments. Our results clearly showed that CD44 knockout mice were resistant to iota toxin versus the parental (CD44^+^) strain, critically reinforcing the role of CD44 during intoxication by the iota-family toxins in diverse cell-based assays and an animal model.

In summary, there are now multiple pieces of evidence that the iota-family toxins from various *Clostridium* species exploit CD44 during the intoxication process. Exactly how CD44 facilitates this remains a focus for future study.

## Materials and Methods

### Ethics Statement

All animal studies were done in accordance with the Virginia Tech Institutional Animal Care and Use Committee via the following approved protocol “Studies into the Receptor for Clostridial Binary Toxins (10-046-TechLab)”. The pain and distress animals experience during handling and injection was momentary. To minimize this, mice were monitored every four hours. Moribund animals would be culled immediately, including those that lose 20% or more of their initial weight. To avoid death as an endpoint, moribund animals would be euthanized using prolonged exposure to carbon dioxide followed by cervical dislocation, with subsequent observation of vital signs for five minutes.

### Reagents

Toxin components from *C. botulinum* (C2), *C. difficile* toxin (CDT), *C. perfringens* (iota), and *C. spiroforme* (CST) were produced and purified as described previously [3], [58]. Recombinant CD44 (standard) fused to IgG was expressed in COS-7 cells and purified as described previously [45]. Purified CD44 (standard) - GST construct was purchased from Abnova.

### Effect of Reducing Agent on Iota Cytotoxicity and Ib Binding

Subconfluent Vero cells were pre-treated for 30 min at 37°C with dithiothreitol (DTT; 5 or 10 mM) in Eagles Minimum Essential Media (EMEM; Invitrogen) containing 10% heat-inactivated fetal bovine serum (FBS). Control cells were incubated in media (with or without DTT) or iota toxin (460 pM Ia +500 pM Ib) alone. At designated time points, pictures were taken to determine the number of rounded cells. Data are given as mean ± S.D. (n = 3). Significance was tested by using Graph Pad Prism 4 and the student’s t-test (* and *** respectively represent p<0.05 and 0.0005; ns = not significant).

Vero cells were incubated for 30 min at 37°C, with or without 10 mM DTT. Cells were cooled on ice, Ib (1 µg/ml EMEM) added, and all incubated together for 30 min at 4°C and subsequently 30 min at 37°C. Cells were lysed in Laemmli sample buffer, heated for 5 min at 95°C, and subjected to SDS-PAGE. Proteins were blotted onto nitrocellulose membrane subsequently blocked with a powdered milk solution. Cell-bound Ib was detected on the nitrocellulose with specific rabbit antibody against Ib, anti-rabbit horseradish-peroxidase conjugate, and the enhanced chemiluminescence (ECL) system. Purified Ib not added to cells was used as a control for the blot.

### Effect of DTT on Enzyme Activity of Ia

The effect of DTT on ADP-ribosyltransferase activity of Ia was tested with Vero cell lysate as previously described by Heine et al. [59]. Ia (22 nM) was pre-treated for 30 min at 4°C with, or without, 10 or 50 mM DTT. Subsequently, Ia (2.2 nM final concentration) was applied to Vero lysate (40 µg total protein) containing 10 µM biotin-NAD^+^ with or without DTT. Controls consisted of lysate and biotin-NAD^+^ without Ia. Samples were incubated at 37°C for 15 min and the enzyme reaction stopped by adding Laemmli sample buffer plus heat (95°C) for 5 min. Following 12.5% SDS-PAGE, proteins were transferred by semi-dry blotting onto a nitrocellulose membrane and the biotinylated (i.e. ADP-ribosylated) G-actin detected by streptavidin-peroxidase and ECL reaction (GE Healthcare). The intensity of biotinylated G-actin was measured by densitometry (Adobe Photoshop 7) and presented as mean ± S.D. (n = 3). Statistical significance was determined by the student’s t-test.

### Inhibition of Iota Cytotoxicity with Anti-CD44 Antibody

Vero cells were plated to confluency in EMEM containing 10% FBS and maintained in a humidified 37°C incubator. Cells were pre-treated with serial dilutions of an anti-CD44 monoclonal antibody (clone IM7 #553133; BD Pharmingen) for 30 min before the addition of Ib and Ia (250 ng/ml each). A non-specific isotype antibody (125 µg/ml) was included as a control. Toxin activity was determined through the binding of phalloidin to F-actin. After 4 h following toxin addition, cells were fixed using a 1∶10 formalin solution for 1 h and permeabilized using 0.1% Triton-X100 in PBS. To visualize the F-actin cytoskeleton, cells were stained with Alexa-488 phalloidin (#A12379; Molecular Probes). Additional staining was done with Hoechst 33342 (#H3570; Molecular Probes) and CellMask Deep Red (#H32721; Molecular Probes) to visualize the nucleus and cytoplasm, respectively. Images were acquired on a Discovery-1 high content imager (Molecular Devices) controlled by MetaXpress software. Integrated intensity values of phalloidin fluorescence represent the mean of nine fields +/− standard deviation. Statistics were done by one way ANOVA with significant differences of p<0.05.

### Cytotoxicity of Clostridial Binary Toxins upon CD44^+^ and CD44^−^ Cells

Human recurrent cutaneous melanoma cells (RPM) naturally devoid of CD44, and those transfected with CD44 (standard) encoding plasmid [24], were subsequently used with varying concentrations of iota-family or C2 toxins. Vero cells provided an additional control. F-actin content was ascertained by staining with Alexa-488 phalloidin after 5 h and “% control” determined versus control cells in media only. Each toxin concentration represents mean +/− standard deviation of duplicate wells from three separate experiments.

### Western Blot and Co-precipitation Analysis of LSR on Cells

Detection of LSR on RPM and Vero cells was done by Western blot using rabbit anti-LSR sera. Initial co-precipitation experiments were done with RPM (CD44^+^ and CD44^−^), as well as Vero, cells. Briefly, cells were grown to confluence in 10 cm dishes. Cells were washed with DMEM and incubated with or without Ib (10^−7^ M) at 37°C for 30 min with medium containing 1% bovine serum albumin. Following PBS washes, cells were subsequently lysed with PBS containing Tris (50 mM, pH 8), NaCl (150 mM), Triton X-100 (0.5%), as well as protease and phosphatase inhibitors. Antibody against CD44 (10 µg) was added to cell lysate (1 ml) at room temperature and rotated for 2 h, followed by protein A beads for 30 min. Beads were centrifuged, washed in PBS, and bound proteins prepared for SDS-PAGE. Following electrophoresis, proteins were transferred onto nitrocellulose and incubated with rabbit anti-LSR sera. There were subsequent serial washings, addition of protein A-horseradish peroxidase conjugate, and then development by ECL.

### Binding of Ib to CD44^+^ and CD44^−^ Cells

Confocal microscopy was done with RPM cells (CD44^+^ vs CD44^−^) incubated for 3 min at 37°C with Cy3-Ib (20 µg/ml), washed with PBS, and then mounted in mowiol. Dapi-stained nuclei are blue.

### Binding of Iota-family B Components to Purified CD44 in Solution

Solution-based experiments were subsequently done using purified CD44 with Ib and other B components from *C. spiroforme* (CSTb), *C. difficile* (CDTb), and *C. botulinum* (C2IIa). B component (10 µg) was added to CD44-IgG or CD44-GST (10 µg) in 20 mM Hepes buffer, pH 7.5 containing 150 mM NaCl for 60 min at room temperature (50 µl total volume). Protein A-agarose (used with CD44-IgG construct) or glutathione-sepharose (used with CD44-GST construct) beads (Sigma) were then added for 5 min at room temperature, gently centrifuged, and washed with buffer. SDS-PAGE sample buffer containing reducing agent was added to the beads, the mixture heated, and protein separated from beads by centrifugation. Supernatant proteins were then separated by 10% SDS-PAGE, transferred onto nitrocellulose, and B components detected with either rabbit anti-Ib or -C2IIa sera (1∶1,000 dilution). Protein A-peroxidase conjugate (Bio-Rad) was used at a 1∶3000 dilution, and following washes, specific B component bands were visualized with SuperSignal West Pico chemiluminescent substrate (Thermo Scientific).

### Mouse Lethality

Homozygous CD44 knockout and wild-type control mice (C57BL/6J parental strain; ∼20 g males) were purchased from Jackson Laboratories [60]. Two separate experiments were done using an intraperitoneal injection of each mouse with sterile PBS containing Ia (0.5 µg) and Ib (0.75 µg). Mice were monitored for morbidity and mortality every 4 h post injection, up to 48 h.
